# SAPrIm 2.0: a semi-automated protocol for mid-throughput soluble HLA immunopeptidomics

**DOI:** 10.3389/fimmu.2025.1546629

**Published:** 2025-04-24

**Authors:** Erwin Tanuwidjaya, Terry C. C. Lim Kam Sian, Joel R. Steele, Gabriel Goncalves, Isaac B. Woodhouse, Janet Chang, Joshua D. Ooi, Ralf B. Schittenhelm, Pouya Faridi

**Affiliations:** ^1^ Department of Medicine, School of Clinical Science, Faculty of Medicine, Nursing & Health Science, Monash University, Clayton, VIC, Australia; ^2^ Monash Proteomics & Metabolomics Platform, Department of Biochemistry and Molecular Biology, Monash Biomedicine Discovery Institute, Monash University, Clayton, VIC, Australia; ^3^ Centre for Cancer Research, Hudson Institute of Medical Research, Clayton, VIC, Australia

**Keywords:** soluble HLA, sHLA, HLA, MHC, immunopeptidomics, plasma immunopeptidomics, mass spectrometry, SAPrIm

## Abstract

Human leukocyte antigen (HLA) molecules are pivotal in guiding human adaptive immune responses through their presentation of peptide ligands, collectively known as the immunopeptidome. This process is central to the development of cancer immunotherapies, such as vaccines and T-cell therapies. Profiling the immunopeptidome from plasma and other biofluids has gained increasing traction, as it offers a minimally invasive approach for monitoring disease states and immune responses toward cancer therapy. Here we present the second iteration of SAPrIm, a refined immunopeptidomics tool optimized for soluble HLA analysis. It can process up to 12 samples per batch within a day. In this plasma-focused iteration, we identified approximately 1,200 to 4,000 immunopeptides from 100 µL to 1 mL of plasma, demonstrating high reproducibility across technical replicates, biological replicates, and inter-day analyses. This robust reproducibility highlights the method’s strong potential for reliable relative quantification of immunopeptides in plasma-based studies. This workflow is positioned to advance the field of immunopeptidomics by enabling efficient plasma-based comparative analyses and mid-size cohort studies.

## Graphical Abstract

Overview of the workflow. 1) Plasma lysis and pre-clearance, 2) Affinity purification of peptide/HLA complex using crosslinked W6/32 to MagReSyn Protein A Max beads, 3) Peptide clean-up, 4) Analysis of the HLA peptides by variable window DIA-MS, 5) Bioinformatics analyses.

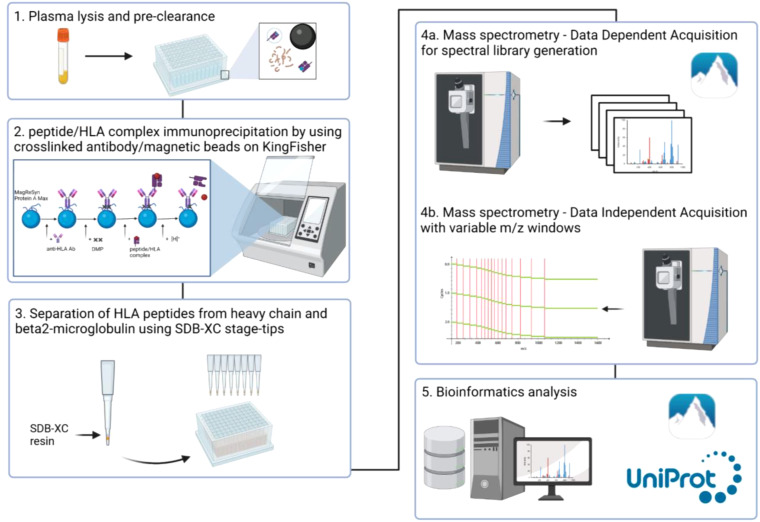

## 1 Introduction

The Human Leukocyte Antigen (HLA) is encoded by the Major Histocompatibility Complex (MHC) genes in humans and is responsible for presenting immunopeptides on the cell surface for T cell recognition (1). These peptides, collectively termed the immunopeptidome, are derived from proteasomal degradation of endogenous (HLA Class I) and exogenous proteins (HLA Class II). Under healthy conditions, the immunopeptidome presented by HLA molecules consists of autologous peptides to which T cells are tolerant (1). However, under pathological circumstances such as infection or cancer, the immunopeptidome contains aberrant peptides that alert T cells of genomic, transcriptomic, and proteomic abnormalities (2).

Although HLA molecules are predominantly expressed on the surface of cells as membrane-bound proteins (mHLA), these proteins can also be found in body fluids as soluble forms (sHLA, 3). These soluble peptide-HLA complexes are thought to originate from cell shedding by membrane metalloproteinase (4) or alternative splicing events (3). However, the precise mechanism of their release has yet to be fully elucidated. Notably, increasing evidence links sHLA to clinical disease progression in cancers (5–7) and in inflammatory conditions (8–10) due to its association with both immune evasion and activation. Despite significant interest, the exploration of the sHLA immunopeptidome has only gained momentum over the past decade, following the landmark study by Bassani-Sternberg et al. (11). They demonstrated up to 86% peptide identification overlap between plasma sHLA and the mHLA peptidome from tumor cells of patients with acute myeloid leukemia and acute lymphoblastic leukemia, validating the strong similarity between the two. Given the close correlation between the immunopeptidomes of sHLA and mHLA, sHLA immunopeptidomics has the potential to complement or even replace traditional mHLA studies (11). Indeed, the promise of sHLA immunopeptidome for clinical applications has been discussed elsewhere (12). Unlike mHLA-based studies, which typically rely on invasive tumor biopsies, sHLA can be analyzed from liquid biopsies, providing a clear benefit for patients such as reduced discomfort and biopsy-associated adverse effects. Recent research has further shown the utility of sHLA immunopeptidomics in profiling tumor-associated antigens from body fluids, which underscores its potential in cancer biomarker discovery and personalized treatment strategies (13, 14).

However, one of the primary challenges in sHLA immunopeptidomics remains the large volume of input material required for analysis—typically milliliters of plasma (14) and even hundreds of milliliters of pleural fluid (13). It is primarily due to the low abundance of sHLA, which necessitates larger sample volumes to achieve sufficient analytical depth. In addition, biofluids contain a high dynamic range of contaminating molecules (such as IgGs, albumin), which masks the detection of these low abundant sHLA molecules (15). The need for large volumes of input material poses challenges for clinical feasibility, particularly for patients from whom obtaining such volumes is difficult. These constraints therefore highlight the need for more efficient, scalable sample preparation techniques that can make sHLA immunopeptidomics feasible for routine clinical use.

We previously developed SAPrIm (Semi-Automated Protocol for mid-throughput Immunopeptidomics, 16), a workflow designed to enhance the efficiency and reproducibility of HLA immunopeptidomics. It was originally designed to work with cells and tissue samples. Here, we have developed a second iteration of our SAPrIm protocol, which we call SAPrIm 2.0, designed for mid-throughput plasma sHLA immunopeptidomics studies. Similar to its predecessor, this protocol can process 12 samples per run and leverages the KingFisher Duo Prime liquid handling system to minimize operator-induced variation and decrease sample preparation time. A data-independent acquisition (DIA) based approach is employed to quantify HLA-bound peptides with high confidence and improve sensitivity and reproducibility (17–21). Ultimately, this approach provides a robust workflow that lowers the barrier of entry to conducting sHLA immunopeptidomics on plasma, advancing the field towards broader clinical applications.

## 2 Materials and methods

### 2.1 Materials

#### 2.1.1 Reagents

- 2-Chloroacetamide (CAA, Sigma #C0267)
- 3-[(3-cholamidopropyl) dimethylammonio]-1-propanesulfonate (CHAPS)(Thermo Fisher Scientific #28300)
- Acetic acid (Sigma #A6283)
- Acetonitrile (ACN, Thermo Fisher Scientific #FSBA955)
- Ammonium Hydroxide (NH4OH) 28% (Sigma #338818)
- Dimethyl pimelimidate (DMP, Sigma #D8388)
- Halt™ Protease and Phosphatase inhibitor (Thermo Fisher Scientific #78442)
- HPLC-grade water (Thermo Fisher Scientific #7732-18-5)
- Hydrochloric acid (HCl) 37% (Sigma #339253)
- Phosphate buffered saline (PBS, Sigma #P5493)
- Sodium Chloride (NaCl, Sigma #S9625)
- Sodium hydroxide (NaOH, Sigma #221465)
- Triethanolamine (TEA, Sigma #90279)
- Trifluoroacetic acid (TFA, Thermo Fisher Scientific #FSBA116)
- Tris hydrochloride (Sigma #10812846001)
- W6/32 Antibody (Leico Technologies #H263)
- LC/MS grade water (Thermo Fisher Scientific #W6-4)

#### 2.1.2 Additional materials/equipment

- Benchtop refrigerated centrifuge 5810R (Eppendorf #EP5811000088)
- Benchtop centrifuge rotor FA-45-30-11 (Eppendorf #EP5427753001)
- Eppendorf LoBind^®^ 1.5 mL tubes (Eppendorf #0030108116)
- Eppendorf LoBind^®^ 2 mL tubes (Eppendorf 0030108132)
- epT.I.P.S^®^ pipette tips (Eppendorf #30073436)
- KingFisher 96 well plate (Thermo Fisher Scientific #95040450)
- KingFisher Duo Prime (Thermo Fisher Scientific #5400110)
- KingFisher tip comb (Thermo Fisher Scientific #97003500)
- MagReSyn^®^ Protein A Max (Resyn Biosciences MR-PAM010)
- Polypropelene Snap Top MS microvial (Thermo Fisher Scientific #6ERV11-03PPCT)
- SDB-XC Solid Phase Extraction disk (CDS Empore™ #13-110-020)
- Snap Ring Seal MS vial cap (Thermo Fisher Scientific #11702428)

### 2.2 Protocol

#### 2.2.1 Crosslinking of antibodies to the magnetic beads (~3 hours)

*Note: This step can be performed during plasma preparation or prepared in advance.*

List of required buffers:

- Crosslinking wash buffer:
  • 200 mM triethanolamine (TEA), pH 8.3
- Dimethyl pimelimidate (DMP) buffer:
  • 5 mM DMP in 200 mM TEA, pH 8.3
- PBS, pH 7
- Tris-HCl 1M, pH 8

MagReSyn preparation (~10 minutes):

1. Resuspend MagReSyn^®^ Protein A MAX beads thoroughly by vortex mixing or inversion to ensure a homogenous suspension.
2. Slowly transfer the beads to a 2 mL Eppendorf LoBind^®^ tube using a pipette.
3. Place the tube on a magnetic separator and allow for beads to clear for approximately 30 seconds. Carefully remove the storage buffer without disturbing the microparticles.
4. Wash the beads in 1 mL volume of PBS 3 times, paying close attention to avoid disturbing the beads in between the washes.
5. Resuspend washed beads in 1 mL volume of PBS. The beads are now ready for antibody binding and pre-clearance. Please refer to **Table 1** for the amount of antibody and beads used in this study.

Antibody/beads crosslinking (~3 hours):

**Table 1:** The ratio of magnetic beads and antibodies used in this protocol per sample.

Plasma	MagReSyn stock (µL)	W6/32 (ug)
500 µL or less	50	100

*Note: Imidoester group on DMP reacts with primary amines to form covalent bonds. Other non-amine buffers suitable as crosslinking wash buffers include PBS and HEPES, pH 8-8.5.*

1. Incubate the prepared beads with anti-HLA Class I (W6/32) antibody (Ab) at 4°C, gently rolling for an hour.
2. Place the tube on a magnetic separator and allow the beads to clear for approximately 30 seconds. Then, remove the supernatant containing any unbound Ab.
3. Wash the beads with 1 mL crosslinking wash buffer three times, paying close attention to avoid disturbing the beads in between washes.
4. Resuspend the washed beads in 100 µL of crosslinking wash buffer. *Note: This volume is suitable for experiments involving 12 samples.*
5. Add 1 mL volume of DMP buffer and incubate at 4°C, gently rolling for an hour.
6. Quench the crosslinking reaction with 122 µL of 1M Tris-HCl, pH 8 to a final concentration of 100 mM, and resume incubation for another 15 minutes to ensure complete quenching.
7. Place the tube on a magnetic separator and allow the beads to clear for approximately 30 seconds. Then, remove the supernatant.
8. Wash the beads with 1 mL PBS 3 times, paying close attention to not disturbing the beads in between washes.
9. Resuspend the washed beads in 300 µL PBS (per sample) and transfer to the KingFisher 96 well plate using pipette.

#### 2.2.2 Plasma lysis and pre-clearance (~2.5 hours)

*Note: This step can be done during antibody/beads crosslinking.*

List of required buffer:

• 10X lysis buffer

 • 7.5% (w/v) CHAPS

 • Halt™ Protease and Phosphatase inhibitor (10X)

 • 100 mM CAA

1. Thaw plasma samples on ice.
2. Clarify the plasma via centrifugation at 20,000 rcf at 4°C for 10 minutes, transferring the supernatant into new 2 mL Eppendorf tubes.
3. Prepare the lysis buffer and keep it cold (0-4°C).
4. Add the 10X lysis buffer into plasma samples to make a final concentration of 1X.
5. Mix gently and incubate at 4°C, rolling for an hour.
6. Clarify the plasma via centrifugation at 20,000 rcf at 4°C for 5 minutes.
7. Add the clarified plasma into a fresh tube containing 50 µL of pre-washed MagReSyn beads. Incubate at 4°C, rolling for 1 hour (pre-clearance step).
8. Place the tube on a magnetic separator and allow for beads to settle for approximately 30 seconds.
9. Transfer the plasma to a KingFisher 96 well plate.

#### 2.2.3 KingFisher plate preparation and immunoaffinity purification (IP, ~3 hours)

**Figure 1:**
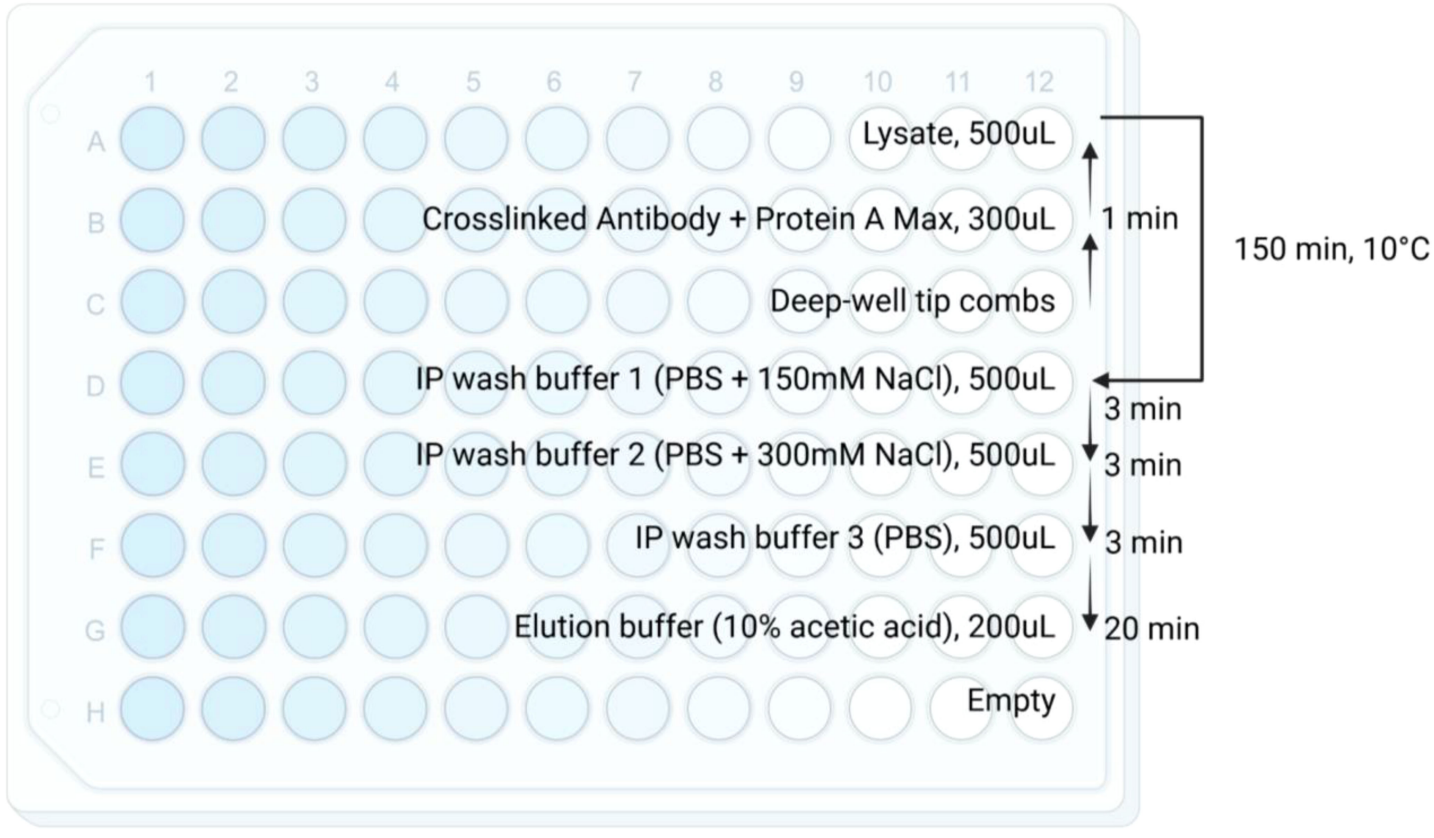
Schematic for KingFisher plate and immunoaffinity purification workflow. Reagents, volumes, and timing are specified in each row. Briefly, crosslinked antibody/magnetic beads are incubated with lysate for 150 minutes at 10°C. The enriched peptide/HLA complexes are then washed with 3 wash buffers and eluted using 10% acetic acid. arrow indicates the direction of the KingFisher protocol. The temperature 10°C is the current lowest temperature limit setting on KingFisher Duo Prime.

List of required buffers:

• IP wash buffer 1

 • 150 mM NaCl in PBS

• IP wash buffer 2

 • 300 mM NaCl in PBS

• IP wash buffer 3

 • PBS

• Elution buffer

 • 10% acetic acid in LC/MS grade water

1. Set up the KingFisher plate according to **Figure 1**. Row A is used for peptide/HLA complex enrichment to maintain constant temperature.
2. KingFisher protocols are summarized in **Figure 1**. Method files can be made available on request.
3. Clarify the eluted peptides via centrifugation at 20,000 rcf for 5 minutes.
4. Transfer to a new set of Eppendorf tubes for storage or load onto SDB-XC material for peptide clean-up (Peptide clean-up, Step 5).

#### 2.2.4 SDB-XC peptide clean-up (~2 hours)

*Note: This step can be performed on a separate day, manually or on plate format via using adapters. If the step is not done immediately, store eluted peptides in -80°C.*

*Note: This step can be replaced with the conventional C18 clean-up for similar performance.*

**Figure 2:**
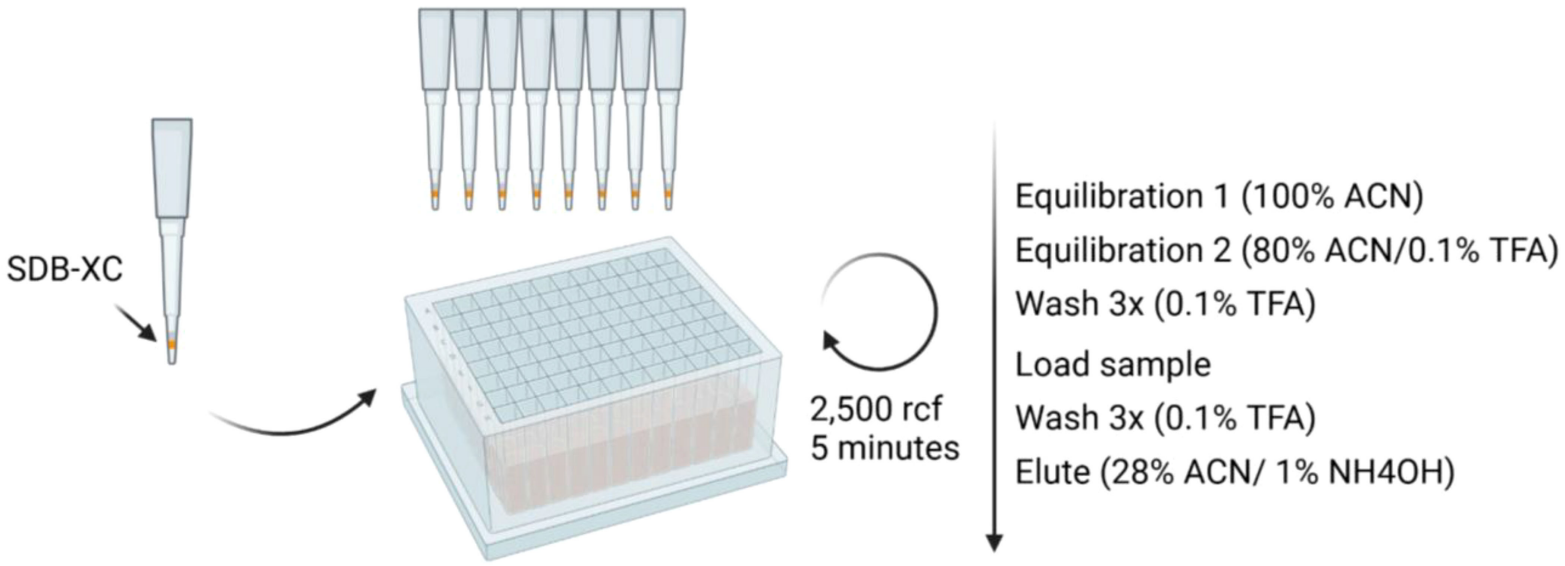
Schematic for SDB-XC peptide clean-up workflow. Reagents and timing are specified. Briefly, premade SDB-XC tips are equilibrated prior to peptide clean-up. Peptides are then loaded onto the tips, washed and eluted.

List of required buffers:

• Equilibration buffer 1

 • 100% ACN

• Equilibration buffer 2

 • 80% ACN/0.1% TFA

• Wash buffer

 • 0.1% TFA

• HLA-I elution buffer

 • 28% ACN/1% NH4OH

1. Set up the stop-and-go-extraction tips (StageTips, 22) by placing 4 punches of SDB-XC SPE material in a p200 pipette tip, according to **Figure 2**.
2. Equilibrate the SDB-XC StageTips with 200 µL of equilibration buffer 1. Spin down the solvent at 2,500 rcf for 5 minutes.
3. Repeat step 2 with the equilibration buffer 2.
4. Repeat step 2 with the wash buffer thrice.
5. Load the sample onto the SDB-XC material. Bind the peptides to the SDB-XC via centrifugation at 2,500 rcf for 5 minutes.
6. Wash the sample 3 times using 200 µL of wash buffer, via centrifugation at 2,500 rcf for 5 minutes.
7. Elute the peptides from the SDB-XC StageTips using 200 µL HLA-I elution buffer into a 1.5 mL Eppendorf tube, via centrifugation at 2,500 rcf for 5 minutes.
8. Dry samples using a vacuum concentrator/lyophilizer.
9. Resuspend the dried peptides in 9 µL of 2% ACN/0.1% TFA.
10. Sonicate the sample for 10 minutes in a water bath sonicator (60Hz, room temperature) to ensure complete resuspension.
11. Clarify the sample using centrifugation, at 20,000 rcf for 5 minutes.
12. Transfer to a mass spectrometry (MS) vial for injection.

#### 2.2.5 Additional methods

##### 2.2.5.1 Mass spectrometry analysis

For MS analysis, peptides were loaded into a trap-and-elute system (Thermo Fisher Scientific Acclaim™ PepMap™ 100 nanoViper, C18, 50 mm x 300 µm, 5 µm, 100 Å for the trap column, and Thermo Fisher Scientific Acclaim™ PepMap™ 100, C18, 50 cm x 75 µm, 2 µm, 100 Å for the analytical column) on an Orbitrap Exploris 480 coupled to an UltiMate 3000 RSLCnano UHPLC system.

MS acquisition settings follow the previously described variable window DIA (vDIA) method on SAPrIm workflow (16). Briefly, peptides were eluted using a 120-minute gradient from 7.5% to 37.5% buffer B (80% ACN/0.1% FA), followed by a 99% buffer B wash for 6 minutes, at a flow rate of 250 µL/min. The MS was operated in data-independent acquisition mode (DIA) using higher-energy collision dissociation (HCD) fragmentation settings. MS1 spectra were acquired from 350 to 1,675 m/z at 120,000 resolution with an RF lens set to 40%. Monoisotopic peak determination was set to peptide with relaxed restriction. Ions were collected to an AGC threshold of 200% and maximum injection time set as auto. For DIA scans, precursors were fragmented using HCD at 27% with a 30,000 resolution. The scan range mode was set to ‘define m/z range’ at 120 to 1,450 m/z. Ions were collected to an AGC threshold of 1000% and injection time set as auto.

For data-dependent acquisition (DDA), peptides were eluted using a 90-minute gradient from 6% to 36% buffer B (80% ACN/0.1% FA), followed by a 99% buffer B wash for 5 minutes, at a flow rate of 250 µL/min. HCD was used for fragmentation. MS1 spectra were acquired from 350 to 1,700 m/z at 120,000 resolution with an RF lens set to 40%. Monoisotopic peak determination was set to peptide with relaxed restriction. Ions were collected to an AGC threshold of 250% or until reaching a maximum injection time of 50 ms. Precursor ion charge was set from +1 to +4, with a dynamic exclusion duration of 10 seconds at 10ppm. For MS2 scans, precursors were isolated using a 1.1 m/z window and fragmented using HCD at 30% with a 15,000 resolution. The scan range mode was set to ‘define first mass’ at 110 m/z. Ions were collected to an AGC threshold of 200% or until reaching a maximum injection time of 100ms.

##### 2.2.5.2 Plasma collection from donor

Plasma was obtained from a healthy donor, following approval of the Monash University Human Research Ethics Committee (HREC number = 13019A). Briefly, blood was withdrawn into EDTA tubes (BD Vacutainer K2EDTA <ns/>367525), mixed by inversion and centrifuged at 1,500 rcf at 4°C for 20 minutes. The plasma was aliquoted, snap-frozen and stored at -80°C. The genomic DNA (gDNA) was used to obtain the donor’s HLA types, using Monarch^®^ gDNA Extraction kit (New England BioLabs #T3010L) according to the manufacturer’s instructions. Subsequently, gDNA was sent to the Australian Red Cross for HLA typing. The HLA alleles of the plasma sample used in this experiment are as follows: HLA-A02:01, HLA-A24:02, HLA-B35:01, HLA-B46:01, HLA-C01:02, HLA-C03:03.

##### 2.2.5.3 Experimental controls

Two types of negative controls were used in this study. The first one involved conducting the entire sample processing workflow without using antibodies during the immunoaffinity purification step (IP control), using 500 µL plasma input volume. The second control involved injecting MS Buffer A (0.1% FA) on the MS (MS blank control) using the same MS acquisition method described previously, prior to sample runs. Here we evaluate the effect of pre-clearance and possible MS-derived contamination. Incorporating a pre-clearance step removes nonspecific binding peptides in the highly complex plasma sample prior to affinity purification, resulting in less contaminating peptides. Similarly, running a MS blank run minimizes the presence of contaminating peptides in the form of carryover from previous runs. While both steps were already implemented in the original SAPrIm protocol, we deem it necessary to include a brief assessment of these controls in the context of processing a highly complex matrix that is plasma. Results are summarized in **Supplementary Table 1** and **Supplementary Figure 1**.

##### 2.2.5.4 Streptavidin-biotin sample preparation

*Note: This section is attached for evaluation purposes only.* Streptavidin-biotin sample preparation closely followed IMBAS-MS (Immunopeptidomics by Biotinylated Antibodies and Streptavidin) publication for 500 µL plasma volume (23). Briefly, plasma samples (500 µL) were incubated overnight with 10 µg of Biotinylated W6/32 Ab (Thermo Fisher Scientific, #13-9983-82) and enriched using magnetic streptavidin beads (ReSyn Bioscience) and washed with 100 µL of 150 mM NaCl in 10 mM Tris pH 8.5, then 100 µL of 450 mM NaCl in 10 mM Tris pH 8.5 and finally 100 µL of 10 mM Tris pH 8.5 at 4°C. The protocol slightly diverged from the publication after affinity purification. In the original publication, the enriched peptides were eluted using 200mM Glycine, pH 2, cleaned using 10 kDa molecular weight cut-off (MWCO) plates (Millipore) and loaded into Evotips Pure for MS injection. In this study, peptides were eluted using 10% acetic acid, subjected to SDB-XC peptide clean-up, and then ran on vDIA MS as described above. These changes were introduced to keep variables outside peptide/HLA complex enrichment constant to allow for better comparison with SAPrIm 2.0.

##### 2.2.5.5 Spectral library generation

A 5mL plasma volume was processed as described above, fractionated into 3 fractions using increasing concentration of ACN during peptide clean-up, and subsequently acquired on DDA-MS to make the DDA experimental spectral library. Raw MS files were loaded into Peaks Studio 12.0 (Bioinformatics Solutions Inc) for spectral library generation with the following settings: precursor mass error tolerance set as 10ppm, fragment mass error tolerance set as 0.02 Da, enzyme set as ‘None’ for unspecific digest mode, max missed cleavage set as 2. N-term acetylation, carbamidomethylation, cysteinylation, deamidation, and methionine oxidation are set as variable modifications. Maximum variable PTM per peptide set as 2.

##### 2.2.5.6 Peptide identification & analysis

Peptide identification search was run on default mode on Peaks Studio 12.0 using the same variable modification settings, against the generated DDA spectral library. The Human Swissprot database (downloaded May 2023) was used as protein inference database for DIA and as database search for DDA. HLA peptide binding prediction analysis was performed using NetMHCpan-4.1 (24) using percentile rank cutoff of ≤2%. Downstream data analysis was performed using R. ANOVA and Tukey’s *post-hoc* test were used for statistical analysis, with P values of <0.05 were considered for statistical significance. Peaks Studio output files for DDA and DIA are available as **Supplementary Table 2**. Immune Epitope Database & Tools (IEDB, 25) were used to check whether the identified peptides have been previously documented.

##### 2.2.5.7 Assessment of inter-assay reproducibility

Duplicates of 500 µL plasma from 3 different healthy donors were processed using SAPrIm 2.0 on two separate days. The study was conducted following the approval of the Monash University Human Research Ethics Committee (HREC number: 13019A). Due to the limited availability of samples, a spectral library was not generated. Consequently, peptide identification was performed using on Peaks Studio 12.0 using DeepNovo Peptidome workflow under default settings, searching against the Human Swissprot database (downloaded May 2023). Sample details and output files are described in **Supplementary Table 3**.

## 3 Results

### 3.1 Protocol design and rationale

The interest in the field of sHLA immunopeptidomics has significantly increased over the past decade. Requirement for large input sample volumes and the lack of reproducibility remain the two most prominent challenges (26). Here we present the second iteration of SAPrIm, which is developed for mid-throughput plasma soluble HLA immunopeptidomics using the KingFisher Duo Prime instrument (Thermo Fisher Scientific).

Several key modifications are incorporated into SAPrIm 2.0 to ensure its suitability for plasma sHLA immunopeptidomics studies. First, an antibody-bead crosslinking step is added to mitigate the interference of plasma soluble IgG antibodies and to minimize co-isolation of non-HLA contaminating peptides. Additionally, SDB-XC material paired with a high-pH elution buffer is employed for peptide clean-up. Importantly, unlike the conventional approach of preparing plasma samples, we include a lysis step prior to affinity purification step, maintaining consistency with the original SAPrIm protocol. We reason that given sHLA might originate from metalloproteinase-mediated cellular shedding (4) and that HLA molecules are present on extracellular vesicles (EVs, 27), peptides presented by the EV-bound HLAs should be taken into consideration as a part of the immunopeptidome (28, 29).

To test this notion, we conducted a proof-of-concept experiment in which we used CHAPS to lyse plasma samples after removing cell debris and larger vesicles by centrifugation. As suspected, we observed a significant increase in peptide identifications (>50% in 0.75% CHAPS, P<0.01), whilst retaining key HLA-I peptide characteristics, such as HLA-I peptide length distribution and proportion of predicted HLA binders (**Supplementary Figure 2**). Although we have yet to elucidate the extent of which vesicle-related peptides add into the plasma immunopeptidome, this observation highlights the benefits of incorporating an additional lysis step in our protocol.

We employed vDIA acquisition method in our original SAPrIm protocol, as DIA has been shown to be a superior alternative to the conventional DDA in proteomics and is increasingly adopted for immunopeptidomics (17, 18). To demonstrate this in the case of SAPrIm 2.0 and in the context of sHLA immunopeptidomics, we compared DDA and vDIA using 500 µL plasma input. In line with our expectation, vDIA significantly outperformed DDA and is able to capture not only the majority of the immunopeptides identified by DDA, but also more peptides (average of 3,530 peptides vs 1,449 peptides, P = 3.31E-4, **Supplementary Figures 3A, B**). Other key peptidome qualities such as the peptide length distribution and peptide binding prediction analysis between the two were otherwise highly similar (**Supplementary Figures 3C, D**).

### 3.2 Qualitative evaluation of SAPrIm 2.0 sHLA peptidome

**Figure 3:**
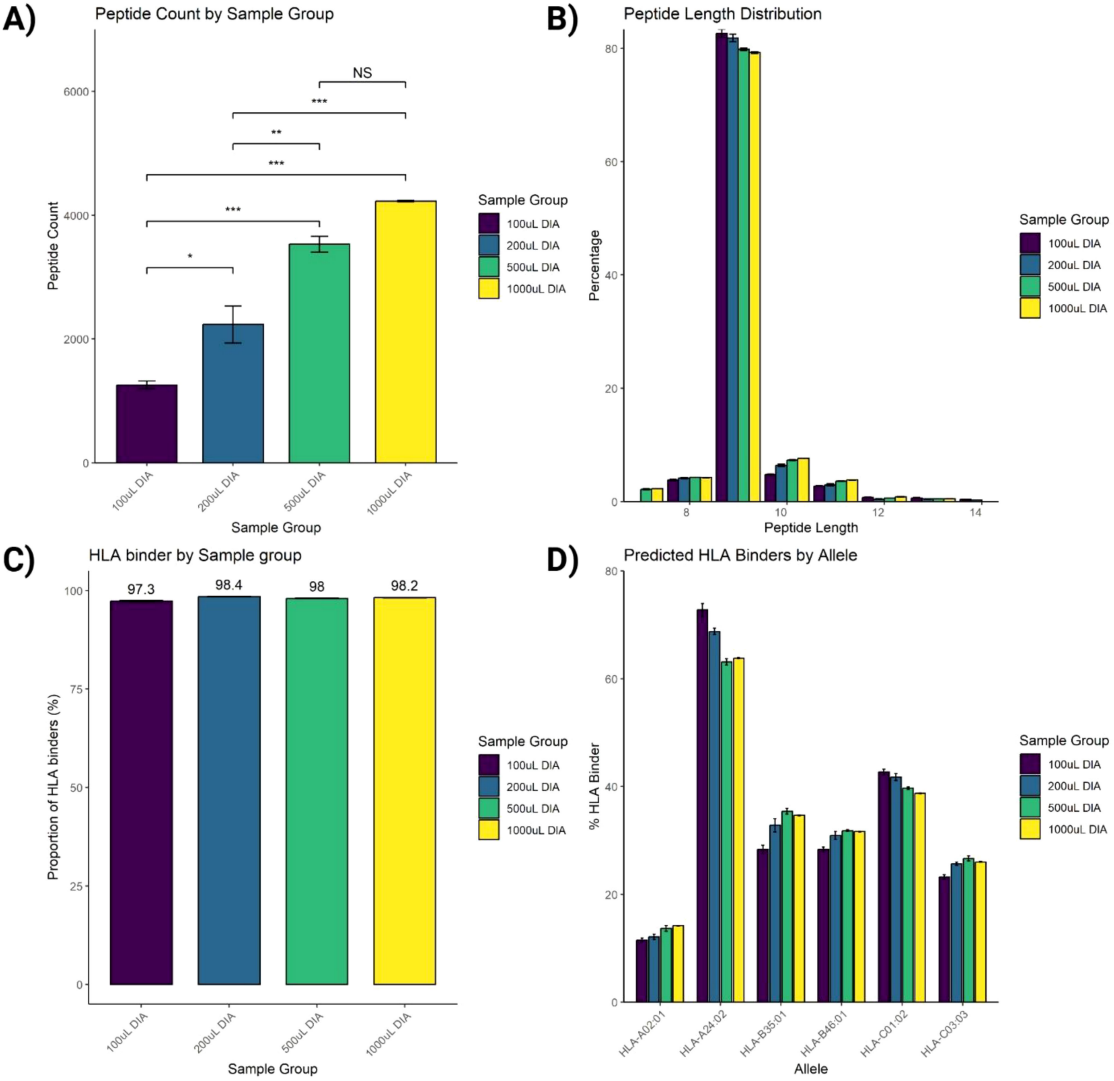
Assessment of immunopeptides peptide identification. **(A)** Increasing the input volume of plasma from 100 µL to 1mL improves the number of identified peptides. **(B)** Length distribution of identified peptides fits the typical HLA-I peptide length distribution. **(C)** Peptide-HLA binding prediction analysis of identified peptides using NetMHCpan analysis tool. **(D)** Proportions of predicted HLA binders by each allele. Data were acquired using 3 replicates and plotted as Mean ± SEM. One-way ANOVA statistical test was conducted with Tukey *post-hoc* analysis, NS, not significant, *P<0.05, **P<0.01, ***P<0.001.

To assess SAPrIm 2.0, we analyzed four plasma input volumes derived from the same plasma pool (100 µL, 200 µL, 500 µL, and 1mL) in triplicates (12 samples total). Based on an underlying spectral library which has been generate in DDA acquisition from 5 mL of fractionate input sample (6,386 search entries), we identified a total of 5,142 unique HLA peptides at a 1% peptide-level false discovery rate (FDR), after correcting for IP and MS blank controls and stripping post-translational modifications (**Supplementary Table 4**). A total of 4,834 (94%) of these peptides are documented in the IEDB databse (accessed on 6th November 2024, **Supplementary Table 4**). The average number of peptides identified from each volume was 1,257 (100 µL), 2,232 (200 µL), 3,530 (500 µL), and 4,226 (1mL, **Figure 3A**). ANOVA and Tukey’s *post-hoc* test showed significant differences between 100 µL vs larger volumes (P<0.01), and between 200 µL and larger volumes (P<0.01). However, the increase between 500 µL and 1 mL was not statistically significant (P = 0.0706, **Figure 3A**).

The peptide length distribution of the identified HLA-I peptides aligned with that of the typical HLA-I peptides, showing a predominant range of 8-12 amino acids (aa), with 9-mers representing the largest proportion (**Figure 3B**). Additionally, binding prediction analysis using NetMHCpan-4.1 on the identified peptides in this HLA-I length distribution showed that over 95% were predicted to bind against the donor HLA allotypes (**Figure 3C**), with its allelic distribution outlined in **Figure 3D**.

**Figure 4:**
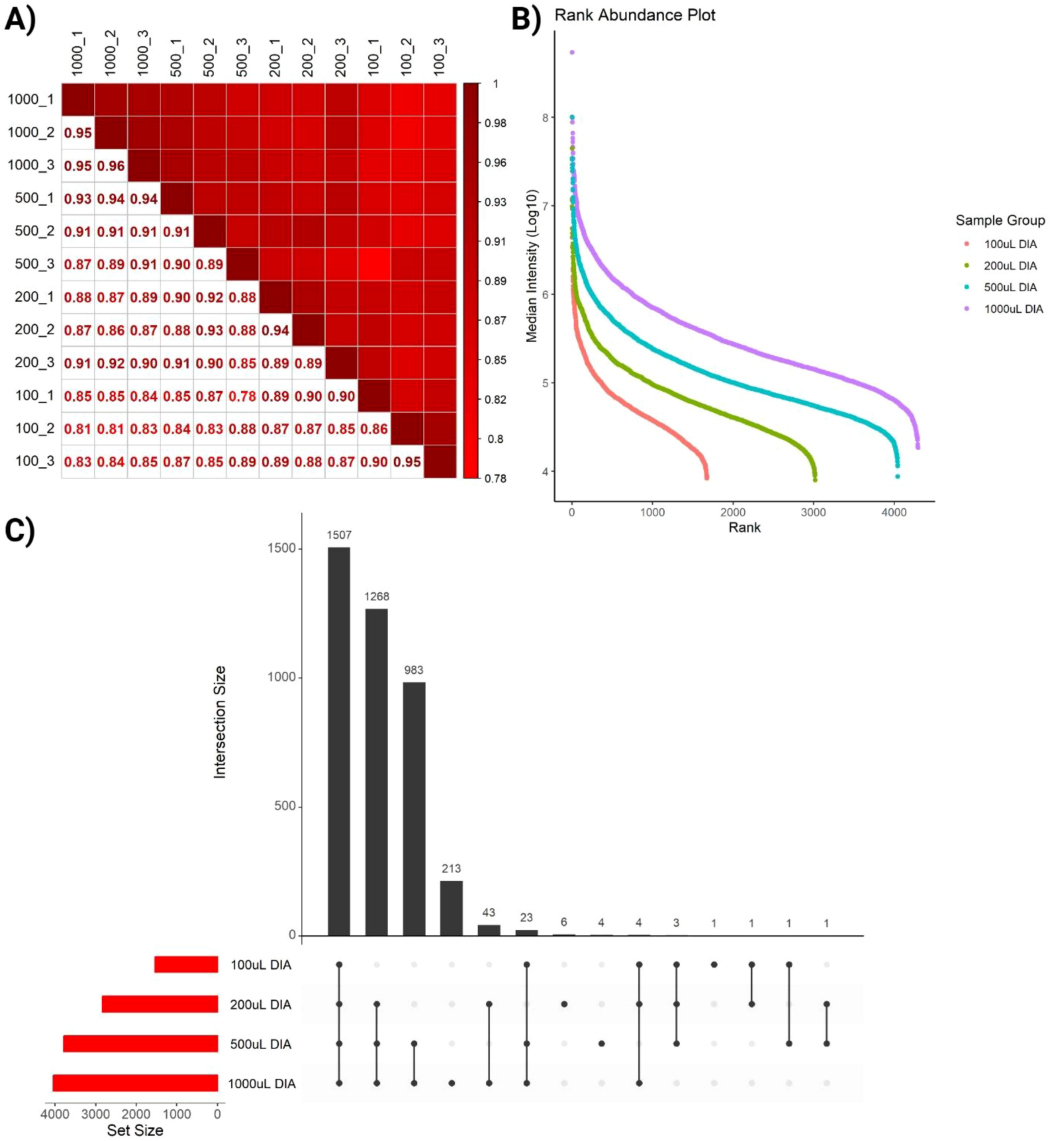
Quality assessment of identified immunopeptides. **(A)** Pearson correlation shows high correlation between replicates across all plasma volumes. **(B)** Intensity Rank plot highlights increased depth as volume increases, represented by increased number of ranks in different sample groups. **(C)** UpSet plot comparing the total number of unique HLA-I peptides from each volume group.

We next sought to evaluate the reproducibility of the SAPrIm 2.0 workflow. Our findings demonstrated its robustness, achieving very high correlation coefficients at the technical replicate levels (Pearson’s correlation, R≥0.90), and between the different sample volumes (R≥0.78, **Figure 4A**). Subsequently, we further examined the workflow’s efficiency in capturing sHLA peptides across increasing plasma volumes, aiming to determine the optimal plasma volume that balances peptide diversity and experimental efficiency. The intensity rank plot (**Figure 4B**) illustrated the diversity of peptides detected at different plasma volumes, where peptides are ranked according to their median intensity. Additionally, the UpSet plot (**Figure 4C**) compared the total number of unique HLA-I peptides from each volume group. The analysis demonstrated a significant increase in peptide identifications with increasing plasma volumes. The 100 µL sample identified 1,540 peptides, and increasing the volume to 200 µL resulted in a gain of 1,293 peptides (83.96%), bringing the total to 2,833 peptides. Increasing further to 500 µL added 957 peptides (33.78%), with a total of 3,790 peptides. Finally, increasing the volume to 1 mL added 251 peptides (6.62%), yielding a total of 4,041 peptides. These findings highlight diminishing returns at higher plasma volumes. While larger volumes improve peptide diversity, the 500 µL sample seems represents an optimal trade-off between maximizing peptide yield and minimizing resource use, including reagent cost and sample availability.

Lastly, we are interested in evaluating the streptavidin/biotin system highlighted in IMBAS-MS (23) to the crosslinked protein A/Ab system outlined in SAPrIm 2.0. Here, triplicate samples were initially processed using streptavidin/biotin system (for 500 µL plasma) up to the sHLA peptide enrichment step and subsequently subjected to SAPrIm 2.0 protocol. In our hand, we found a better performance of crosslinked protein A/Ab system over streptavidin/biotin in the peptidome coverage (3,530 peptides vs 1,361 peptides, P = 1.31E-3, **Supplementary Figure 4A**). Other key peptidome qualities such as the peptide length distribution and peptide binding prediction analysis were otherwise highly similar (**Supplementary Figures 4B, C**), demonstrating an excellent capability of both approaches to address the complexity of sHLA peptide extraction from plasma. We acknowledge that the streptavidin-biotin system adapted from IMBAS protocol might not be fully optimized in our laboratory and therefore might be subjected to artifacts. Additionally, we do not have access to the custom-ordered biotinylated antibodies outlined in the original publication, which performance may not be entirely replicable using commercially available alternatives.

### 3.3 Quantitative evaluation of SAPrIm 2.0 sHLA peptidome

**Figure 5:**
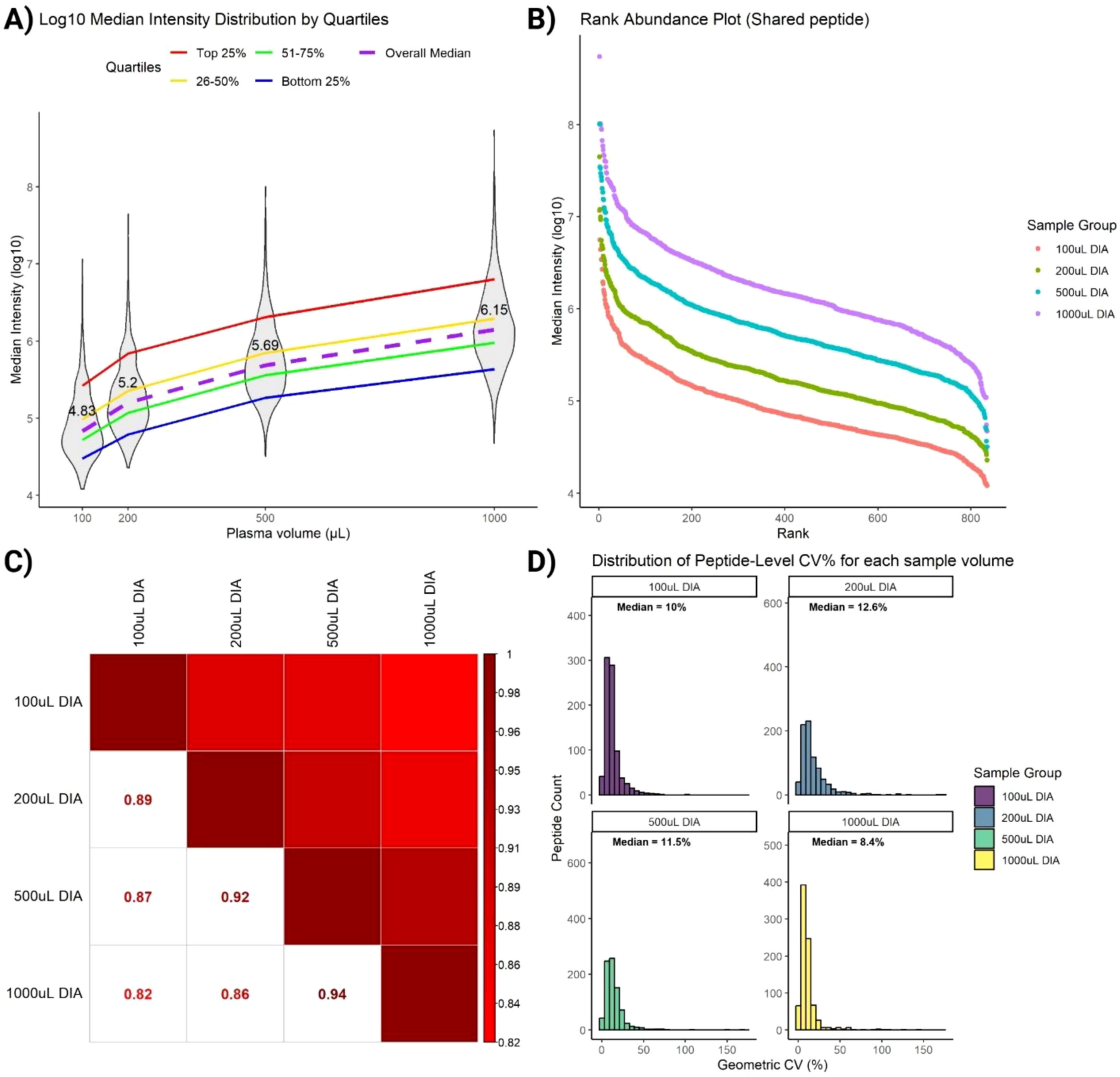
Assessment of immunopeptides commonly identified in all DIA samples. **(A)** Violin plot shows non-linear increase of peptides intensities as plasma volume increases (median labelled). Peptides are stratified on the ranking into 4 quartiles. **(B)** Rank Intensity plot of commonly identified peptides highlights increase of peptide intensities for each peptide rank. **(C)** Spearman rank correlation analysis retains similarities between peptides across sample volumes. **(D)** Coefficient of variation (CV) plot of commonly identified peptides indicates low variabilities (median CV < 13%) for all sample volumes.

To evaluate the quantitative performance of SAPrIm 2.0, we further interrogated a panel of immunopeptides (peptides n = 835) consistently identified across all DIA samples for quantification (conditions and replicates). These peptides are of interest as they represent the core immunopeptidome captured in this study and serve as a benchmark for assessing the method’s recovery efficiency across increasing plasma volumes. A non-linear increase in median peptide intensity was observed as plasma volume increased, with median fold changes of 2.3-fold between 100 µL and 200 µL, 7.2-fold between 100 µL and 500 µL, and 20.9-fold between 100 µL and 1 mL (**Figure 5A**).

To gain deeper insights into peptide recovery trends, peptides were ranked and stratified into quartiles based on their median intensities. Stratification revealed distinct recovery behaviors across plasma volumes. High-abundance peptides (top 25% quartile) exhibited a near-linear increase in log10-intensity with increasing plasma volume, reflecting efficient and reproducible recovery of these peptides (**Figure 5A**, red line). This trend underscores the robustness of SAPrIm 2.0 in capturing high-abundance peptides, with their recovery benefiting significantly from larger sample inputs. In contrast, low-abundance peptides (bottom 25% quartile) showed a markedly weaker increase in intensity with increasing plasma volume (**Figure 5A**, blue line). These peptides demonstrated limited recovery efficiency even at higher plasma volumes, likely reflecting challenges in detecting peptides near the limit of detection. Intermediate-abundance peptides (26–50% and 51–75% quartiles) exhibited more moderate trends (**Figure 5A**, yellow and green lines). Despite variability among quartiles, the overall median (**Figure 5A**, purple dashed line) showed a consistent increase with plasma volume. This suggests that while high-abundance peptides benefit most from increased sample input, overall recovery is influenced by peptide abundance and method sensitivity.

To further assess the robustness of SAPrIm 2.0, we analyzed changes in peptide abundance ranks across plasma volumes. The fold-change in intensities is visualized in the upward shift of the rank intensity plot (**Figure 5B**). Spearman’s rank correlation test showed high similarity in peptide rank distributions between volume groups, with R² = 0.94 between 500 µL and 1000 µL, and R² = 0.82 between 100 µL and 1000 µL (**Figure 5C**). Supporting this, the Coefficient of Variation (CV) plot on peptide intensities shows consistent median CV values below 13% on each plasma volumes (**Figure 5D**). This demonstrate that the peptide abundances of the core immunopeptidome remain highly consistent despite differences in plasma input, further supporting the reproducibility of SAPrIm 2.0.

### 3.4 Inter-assay reproducibility evaluation of SAPrIm 2.0

To determine the day-to-day consistency of SAPrIm 2.0, we compared the immunopeptidomes of three healthy donors (**Supplementary Table 3**) collected on two separate days. Across donors, the total number of identified peptides, their length distributions, and the proportions of predicted binder peptides remained highly stable between timepoints (**Supplementary Figures 5A-C**). Pairwise Pearson’s correlation of peptide intensities supported this finding, demonstrating strong concordance at both the technical replicate level (R ≥ 0.89) and the timepoint level (R ≥ 0.84) for samples from the same donor (**Supplementary Figure 5D**). In contrast, samples from different donors exhibited only moderate correlations (R ~ 0.1–0.8), suggesting that the majority of variation arises from true biological differences rather than technical imprecision. Principal component analysis (PCA) further highlighted the robustness of the assay, as samples from each donor clustered tightly while remaining distinct from other donors (**Supplementary Figure 5E**). Notably, Donors 1 and 3 displayed higher similarity, potentially reflecting their shared HLA-A24:02 allele. Overall, these results confirm that SAPrIm 2.0 maintains strong inter-day assay reproducibility while reliably capturing donor-specific immunopeptidome profiles.

## 4 Discussion

Here we present SAPrIm 2.0, a semi-automated sHLA protocol for low input plasma volume. This protocol has seen the implementation of key modifications to address the challenges of analyzing the highly complex plasma samples and to maximize the peptidome coverage. In particular, crosslinking the antibodies to the beads and implementing a lysis step has had a tremendous effect on the efficiency of the protocol. Similar to the previous iteration, we leveraged vDIA acquisition to increase the depth of the peptidome coverage, whilst lowering the number of missing values. Using SAPrIm 2.0, we were able to identify between 1,257 and 4,226 HLA-I peptides from 100 µL to 1 mL plasma.

Importantly, this protocol is highly reproducible at intra- and inter-assay level, which is critical for clinical applications. It improves confidence in data quality where sample volume is often limited and not sufficient for technical replicates. The KingFisher instrument utilizes a programmable magnetic bead-based workflow that synergizes with the standardized protocol described above, ensuring uniform processing conditions for each sample across different experiments. In larger experiments, a designated sample well could be dedicated to an inter-assay control, using pooled plasma or common reference samples stored in the laboratory.

Flexibility remains one of the key focuses of SAPrIm protocol. While this study leverages the KingFisher automation system for enhanced reproducibility and efficiency, the protocol is equally compatible with other magnet-based platforms. Researchers also have the option to manually perform the workflow using magnetic racks, providing accessibility to laboratories without automated systems, albeit with potentially increased variability and labor intensity. The immunopeptidomics data generated in this study was searched against an experimental-specific spectral library generated from DDA acquisition of higher plasma volume, as often seen in the conventional DIA-based experiment. Alternatively, researchers have the options to do library-free search (30) or other spectral library-based approach i.e. pan-library (31) or deep learning-aided spectral libraries (32, 33), depending on the specific requirements of their studies. While this study focuses on the mid-throughput applications, this protocol can be seamlessly scaled up to simultaneously process 96 samples using the KingFisher Apex instrument (Thermo Fisher Scientific) for larger experiments.

Although this study primarily focuses on plasma, this protocol is applicable for serum or other body fluids sHLA immunopeptidomics studies. Notably, Ritz et al. have previously reported that the sHLA immunopeptidome profiles of plasma and serum exhibited significant similarities, as shown in samples taken from 3 different donors (34). Despite the similarities, there are some key considerations for serum sHLA immunopeptidomics. The key distinction between these biofluids is the absence of clotting factors in serum. Serum preparation involves allowing blood to clot, which can result in a net loss of proteins due to entrapment of proteins within the fibrin clot. Additionally, the activation of proteases in the coagulation process may lead to protein modifications and degradations, as shown empirically in proteomics studies (35, 36). Therefore, we expect lower peptidome coverage in serum for the same amount of volume used to analyze its plasma counterpart.

## 5 Conclusion & future directions

In conclusion, SAPrIm 2.0 provides a robust, efficient, and accessible workflow that bridges the gap between research and clinical translational immunopeptidomics, enabling mid-throughput analysis with short turnover times. By incorporating a few key changes, we have made this protocol suitable for blood-derived biofluids. Future applications of this workflow include extending its utility to investigate the clinical significance of HLA-bound peptides in biofluids beyond blood and its derivatives, where the presence of HLA complexes is well-established (12, 13). Expanding the SAPrIm methodology series to diverse biofluids presents unique challenges, such as optimization of high sensitivity sample preparation methods, HLA enrichment and MS instrumentation. Variations in protein composition, viscosity, and the presence of interfering substances across different biofluids may require tailored adjustments to the SAPrIm 2.0 protocol. For instance, reduced protein levels in certain biofluids could necessitate higher input volumes, and more sensitive mass spectrometers may be needed to achieve greater depth of immunopeptidome coverage. Further refinements to the affinity purification steps can also help extend the use of SAPrIm-based approaches to a broader range of biofluid matrices. Moreover, exploring SAPrIm 2.0 in disease-relevant samples—such as plasma from oncology or autoimmune cohorts—has strong potential for advancing clinical validation and expanding the utility of this platform.

## 6 Troubleshooting

**Table:** 

Problem	Possible reason	Solution
Beads clumping after crosslinking	Insufficient beads resuspension Magnetic attraction between beads	Ensure continuous mixing during crosslinking
Low peptide yield	Reagents stability Antibody performance Buffer pH Mass spectrometer performance Sample loss during clean-up due to adsorption to the tubes Sample loss due to aggregation to the beads	Check the reagents and parameters in the workflow. They are critical and should always be tested prior to any experiment. Ensure the usage of lo-bind tubes to minimize peptide binding to the wall of the tubes. Clarify the eluted peptides from the residual beads prior to storage if peptide clean-up step is conducted the next day.
Poor reproducibility	Uneven distribution of beads/antibody complexes in different samples	Ensure thorough homogenization and dispensation of equal volumes of crosslinked beads in each sample prior to affinity purification step.
Low fraction of predicted peptide binders	Mass spectrometer performance Contamination during enrichment	Ensure blank run is done to remove carryover contaminations. Perform quality assessment of mass spectrometer performance prior to sample run. Ensure thorough washes during affinity purification

## Data Availability

The names of the 515 repositry/repositories and accession number(s) can be found below: ProteomeXchange Consortium via the 516 PRIDE (37) partner repository with the dataset identifier PXD058880.

## Supplementary material

Supplementary Figure 1 Quality assessment of peptides identified in no antibody IP control. **(A)** Length distribution of identified peptides does not follow the typical HLA-I peptide length distribution. **(B)** A total of 4 (5.7%) out of 70 identified peptides are predicted to be HLA binders by NetMHCpan-4.1.

Supplementary Figure 2 Assessment of lysis buffer on plasma sample preparation. **(A)** Peptide identification increases after lysis step and with higher CHAPS percentage, **(B)** Identified peptides follow the typical HLA-I peptide length distribution **(C)** Binding prediction analysis of identified peptides using NetMHCpan-4.1 shows high quality immunopeptidome for all conditions. Data were acquired using DDA-MS on duplicate samples and plotted as Mean ± SEM. One-way ANOVA statistical analysis with Tukey’s *post-hoc* analysis was used to test the sample differences. NS = not significant, *P<0.05, **P<0.01. Note: One replicate was used for 0.75% CHAPS sample group due to technical issues.

Supplementary Figure 3 Peptidome comparison of DIA and DDA on 500 µL plasma. **(A)** vDIA outperforms DDA in peptide identification. **(B)** DIA captures most of the peptides identified in DDA., in addition to significantly more peptides. **(C)** Length distribution of identified peptides in each group follows the typical HLA-I peptide length distribution. **(D)** NetMHCpan4.1 binding analysis. Data were acquired using 3 replicates and plotted as Mean ± SEM. Unpaired t-test was conducted to test for statistical differences in peptide count. ***P <0.001.

Supplementary Figure 4 Immunopeptidome comparison of SAPrIm 2.0 and streptavidin-biotin system on 500 µL plasma. **(A)** Crosslinking antibody/beads system employed in SAPrIm 2.0 outperforms streptavidin-biotin system. **(B)** Peptide Length Distribution of identified peptides. **(C)** NetMHCpan4.1 binding analysis. Unpaired t-test statistical analysis was performed to test for statistical significance. ** P<0.01.

Supplementary Figure 5 Inter-assay evaluation of SAPrIm 2.0 on 500 µL plasma. **(A)** Peptide identification, (**B)** Peptide length distribution, and (**C**) Proportion of HLA binder by NetMHCpan-4.1 remains stable across two separate experimental runs. (**D)** Pearson’s correlation analysis on peptide intensity shows high correlation between samples sourced from the same donor across 2 different timepoints, but modest correlation between samples sourced from different donors. (**E)** Principal component analysis shows tight clustering of immunopeptidome sourced from the same donors, and highlights distinct separation of immunopeptidome from different donors.

## References

[1] Rock KL Reits E Neefjes J . Present yourself! By MHC class I and MHC class II molecules. Trends Immunol. (2016) 37:724–37. doi: 10.1016/j.it.2016.08.010 PMC5159193 27614798

[2] Mosaad YM . Clinical role of human leukocyte antigen in health and disease. Scand J Immunol. (2015) 82:283–306. doi: 10.1111/sji.12329 26099424

[3] Tabayoyong WB Zavazava N . Soluble HLA revisited. Leuk Res. (2007) 31:121–5. doi: 10.1016/j.leukres.2006.06.008 PMC1876709 16860865

[4] Demaria S Bushkin Y . Soluble HLA proteins with bound peptides are released from the cell surface by the membrane metalloproteinase. Hum Immunol. (2000) 61:1332–8. doi: 10.1016/S0198-8859(00)00213-5 11163090

[5] Shimura T Hagihara M Yamamoto K Takebe K Munkhbat B Ogoshi K . Quantification of serum-soluble HLA class I antigens in patients with gastric cancer. Hum Immunol. (1994) 40:183–6. doi: 10.1016/0198-8859(94)90067-1 7960961

[6] Albitar M Vose J Johnson M Do K Day A Jilani I . Clinical relevance of soluble HLA-I and β2-microglobulin levels in non-Hodgkin’s lymphoma and Hodgkin’s disease. Leuk Res. (2007) 31:139–45. doi: 10.1016/j.leukres.2006.02.013 16545870

[7] Wierenga APA Gezgin G van Beelen E Eikmans M Spruyt-Gerritse M Brouwer N . Soluble HLA in the aqueous humour of uveal melanoma is associated with unfavourable tumour characteristics. Cancers (Basel). (2019) 11:1202. doi: 10.3390/cancers11081202 31426578 PMC6721510

[8] Tsuchiya N Shiota M Yamaguchi A Ito K . Elevated serum level of soluble HLA class I antigens in patients with systemic lupus erythematosus. Arthritis Rheum. (1996) 39:792–6. doi: 10.1002/art.1780390511 8639176

[9] Verbruggen LA Versaen H Rebmann V Duquet W De Cock S Grosse-Wilde H . Soluble HLA-DR levels in serum are associated with therapy and genetic factors in rheumatoid arthritis. Hum Immunol. (2002) 63:. 758–64. doi: 10.1016/s0198-8859(02)00431-7 12175730

[10] Wiendl H Feger U Mittelbronn M Jack C Schreiner B Stadelmann C . Expression of the immune-tolerogenic major histocompatibility molecule HLA-G in multiple sclerosis: implications for CNS immunity. Brain. (2005) 128:2689–704. doi: 10.1093/brain/awh609 16123145

[11] Bassani-Sternberg M Barnea E Beer I Avivi I Katz T Admon A . Soluble plasma HLA peptidome as a potential source for cancer biomarkers. Proc Natl Acad Sci. (2010) 107:18769–76. doi: 10.1073/pnas.1008501107 PMC2973870 20974924

[12] Tanuwidjaya E Schittenhelm RB Faridi P . Soluble HLA peptidome: A new resource for cancer biomarkers. Front Oncol. (2022) vol:1069635. doi: 10.3389/fonc.2022.1069635 36620582 PMC9815702

[13] Khazan-Kost S Cafri G Melamed Kadosh D Mooshayef N Chatterji S Dominissini D . Soluble HLA peptidome of pleural effusions is a valuable source for tumor antigens. J Immunother Cancer. (2022) 10:e003733. doi: 10.1136/jitc-2021-003733 35580925 PMC9114951

[14] Shraibman B Barnea E Kadosh D Haimovich Y Slobodin G Rosner I . Identification of tumor antigens among the HLA peptidomes of glioblastoma tumors and plasma. Mol Cell Proteomics. (2019) 18:1255–68. doi: 10.1074/mcp.RA119.001524 PMC6553928 31154438

[15] Anderson NL Anderson NG . The human plasma proteome. Mol Cell Proteomics. (2002) 1:845–67. doi: 10.1074/mcp.R200007-MCP200 12488461

[16] Lim Kan TCC Golcalves G Steele JR Shamekhi T Bramberger L Jin D . SAPrIm, a semi-automated protocol for mid-throughput immunopeptidomics. Front Immunol. (2023) 14:1107576. doi: 10.3389/fimmu.2023.1107576 37334365 PMC10272402

[17] Dowell JA Wright LJ Armstrong EA Denu JM . Benchmarking quantitative performance in label-free proteomics. ACS Omega. (2021) 6:2494–504. doi: 10.1021/acsomega.0c04030 PMC7859943 33553868

[18] Fröhlich K Fahmer M Brombacher E Seredynska A Maldacker M Kreutz C . Data-independent acquisition: A milestone and prospect in clinical mass spectrometry-based proteomics. Mol Cell Proteomics. (2024) 23:100800. doi: 10.1016/j.mcpro.2024.100800 38880244 PMC11380018

[19] Shahbazy M Ramarathinam S Illing P Jappe E Faridi P Croft N . Benchmarking bioinformatics pipelines in data-independent acquisition mass spectrometry for immunopeptidomics. Mol Cell Proteomics. (2023) 22(4):100515. doi: 10.1016/j.mcpro.2023.100515 36796644 PMC10060114

[20] Faridi P Aebersold R Caron E . A first dataset toward a standardized community-driven global mapping of the human immunopeptidome. Data Brief. (2016) 7:201–5. doi: 10.1016/j.dib.2016.02.016 PMC4773481 26958639

[21] Son ET Faridi P Paul-Heng M Leong M English K Ramarathina S . The self-peptide repertoire plays a critical role in transplant tolerance induction. J Clin Invest. (2021) 131. doi: 10.1172/JCI146771 PMC8553557 34428180

[22] Rappsilber J Mann M Ishihama Y . Protocol for micro-purification, enrichment, pre-fractionation and storage of peptides for proteomics using StageTips. Nat Protoc. (2007) 2:1896–906. doi: 10.1038/nprot.2007.261 17703201

[23] Wahle M Thielert M Zwiebel M Skowronek P Zeng W-F Mann M . IMBAS-MS discovers organ-specific HLA peptide patterns in plasma. Mol Cell Proteomics. (2024) 23:100689. doi: 10.1016/j.mcpro.2023.100689 38043703 PMC10765297

[24] Reynisson B Alvarez B Paul S Peters B Nielsen M . NetMHCpan-4.1 and NetMHCIIpan-4.0: Improved predictions of MHC antigen presentation by concurrent motif deconvolution and integration of MS MHC eluted ligand data. Nucleic Acids Res. (2020) W449–454. doi: 10.1093/NAR/GKAA379 PMC7319546 32406916

[25] Vita R Overton J Greenbaum J Ponomarenko J Clark J Cantrell J . The immune epitope database (IEDB) 3.0. Nucleic Acids Res. (2015) 43:D405–12. doi: 10.1093/nar/gku938 PMC4384014 25300482

[26] Caron E Aebersold R Banaei-Esfahani A Chong C Bassani-Sternberg M . A case for a human immuno-peptidome project consortium. Immunity. (2017) 47:203–8. doi: 10.1016/j.immuni.2017.07.010 28813649

[27] van Niel G D’Angelo G Raposo G . Shedding light on the cell biology of extracellular vesicles. Nat Rev Mol Cell Biol. (2018) 19:213–28. doi: 10.1038/nrm.2017.125 29339798

[28] Bauzá-Martinez J Heck AJR Wu W . HLA-B and cysteinylated ligands distinguish the antigen presentation landscape of extracellular vesicles. Commun Biol. (2021) 4:825. doi: 10.1038/s42003-021-02364-y 34211107 PMC8249458

[29] Boyne C Lennox D Beech O Powis SJ Kumar P . What is the role of HLA-I on cancer derived extracellular vesicles? Defining the challenges in characterisation and potential uses of this ligandome. Int J Mol Sci. (2021) 22. doi: 10.3390/ijms222413554 PMC8703738 34948350

[30] Tsou C-C Avtonomov D Larsen B Tucholska M Choi H Gingras A . DIA-Umpire: comprehensive computational framework for data-independent acquisition proteomics. Nat Methods. (2015) 12:258–64. doi: 10.1038/nmeth.3255 PMC4399776 25599550

[31] Pak HS Michaux J Huber F Chong C Stevenson B Müller M . Sensitive immunopeptidomics by leveraging available large-scale multi-HLA spectral libraries, data-independent acquisition, and MS/MS prediction. Mol Cell Proteomics. (2021) vol. 20. doi: 10.1016/J.MCPRO.2021.100080 PMC8724634 33845167

[32] Gessulat S Schmidt T Zolg D Samaras P Schnatbaum K Zerweck J . Prosit: proteome-wide prediction of peptide tandem mass spectra by deep learning. Nat Methods. (2019) 16:509–18. doi: 10.1038/s41592-019-0426-7 31133760

[33] Zeng W-F Zhou X Willems S Ammar C Wahle M Bludau I . AlphaPeptDeep: a modular deep learning framework to predict peptide properties for proteomics. Nat Commun. (2022) 13:7238. doi: 10.1038/s41467-022-34904-3 36433986 PMC9700817

[34] Ritz D Gloger A Neri D Fugmann T . Purification of soluble HLA class I complexes from human serum or plasma deliver high quality immuno peptidomes required for biomarker discovery. Proteomics. (2017) 17:1600364. doi: 10.1002/pmic.201600364 PMC5557337 27862975

[35] Espinosa C Ali S Khan W Khanam R Pervin J Price J . Comparative predictive power of serum vs plasma proteomic signatures in feto-maternal medicine. AJOG Global Rep. (2023) 3:100244. doi: 10.1016/j.xagr.2023.100244 PMC10339042 37456144

[36] Zimmerman LJ Li M Yarbrough WG Slebos RJC Liebler DC . Global stability of plasma proteomes for mass spectrometry-based analyses. Mol Cell Proteomics. (2012) 11:M111.014340. doi: 10.1074/mcp.M111.014340 PMC3433892 22301387

[37] Perez-Riverol Y Bai J Bandla C Garcia-Seisdedos D Hewapathirana S Kamatchinathan S . The PRIDE database resources in 2022: a hub for mass spectrometry-based proteomics evidences. Nucleic Acids Res. (2022) 50:D543–52. doi: 10.1093/nar/gkab1038 PMC8728295 34723319

