# Supplementary material for: SAPrIm 2.0: a semi-automated protocol for mid-throughput soluble HLA immunopeptidomics
Source: Front Immunol. 2025 Apr 24;16:1546629. doi: 10.3389/fimmu.2025.1546629 (PMC12058715; doi:10.3389/fimmu.2025.1546629)
Supplement: Supplementary Figure 1 — Quality assessment of peptides identified in no antibody IP control. (A) Length distribution of identified peptides does not follow the typical HLA-I peptide length distribution. (B) A total of 4 (5.7%) out of 70 identified peptides are predicted to be HLA binders by NetMHCpan-4.1. [file DataSheet1.docx]

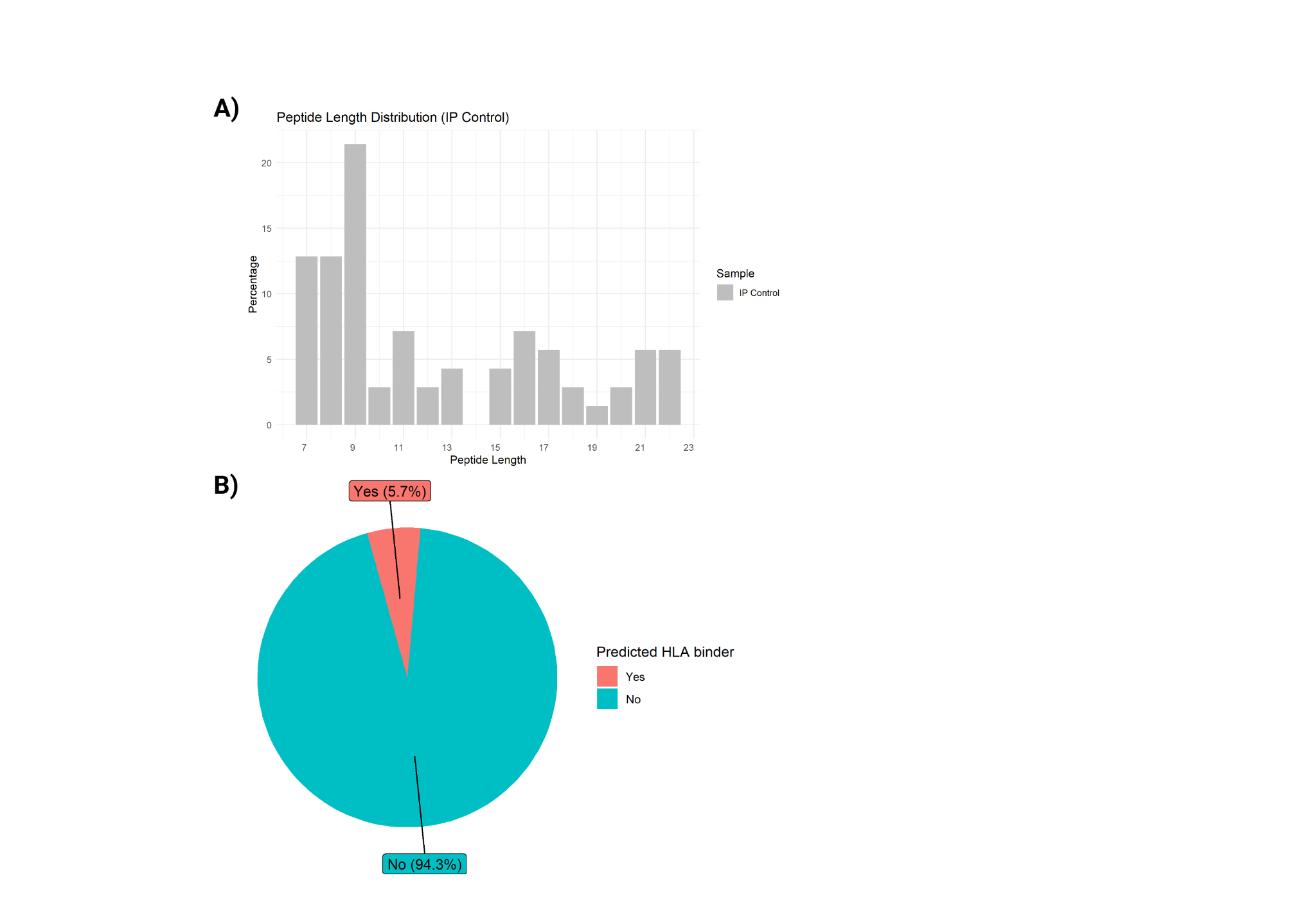


**Supplementary Figure 1. Quality assessment of peptides identified in no antibody IP control.** **A)** Length distribution of identified peptides does not follow the typical HLA-I peptide length distribution. **B)** A total of 4 (5.7%) out of 70 identified peptides are predicted to be HLA binders by NetMHCpan-4.1.


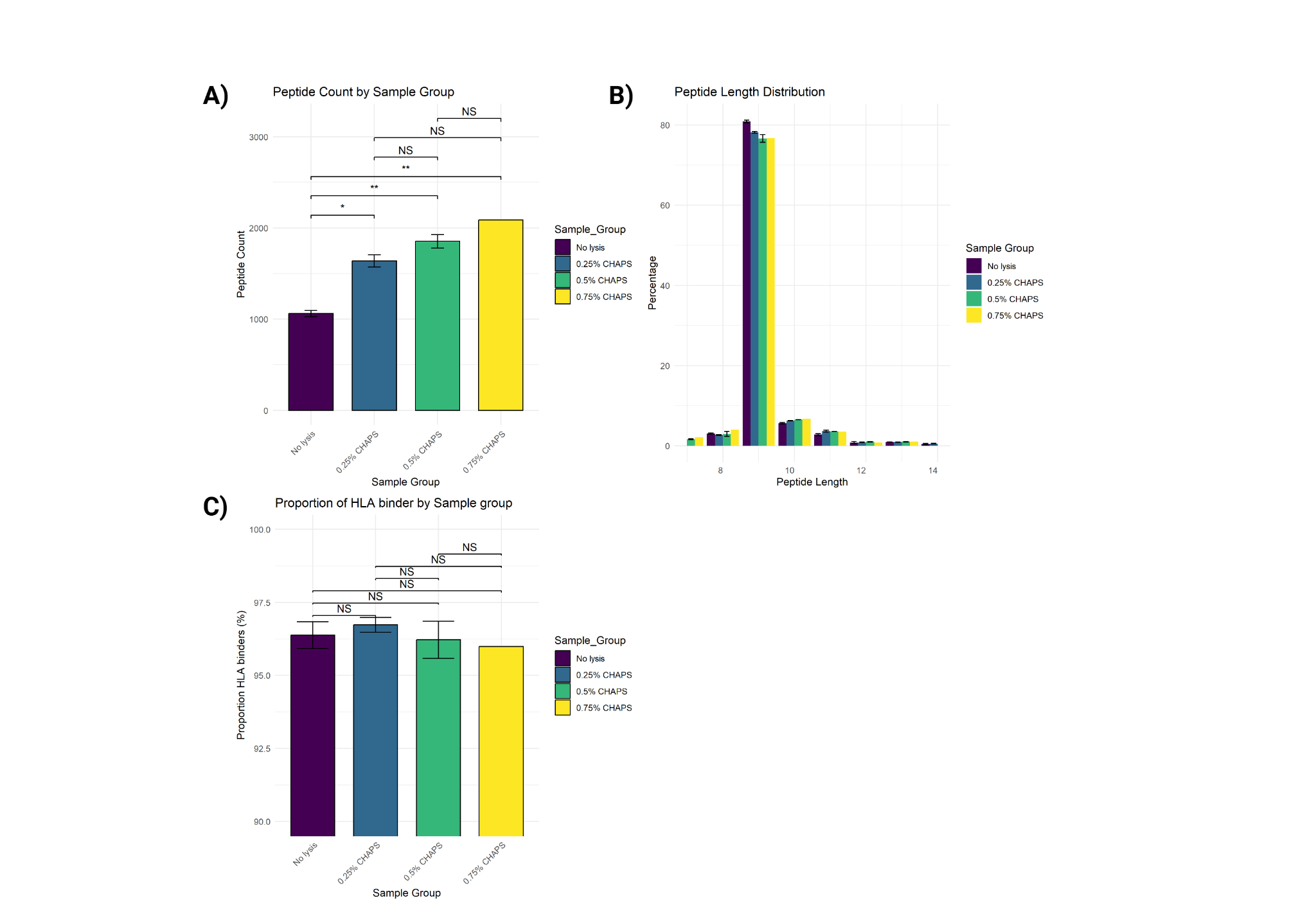


**Supplementary Figure 2. Assessment of lysis buffer on plasma sample preparation.** A) Peptide identification increases after lysis step and with higher CHAPS percentage, B) Identified peptides follow the typical HLA-I peptide length distribution C) Binding prediction analysis of identified peptides using NetMHCpan-4.1 shows high quality immunopeptidome for all conditions. Data were acquired using DDA-MS on duplicate samples and plotted as Mean ± SEM. One-way ANOVA statistical analysis with Tukey’s post-hoc analysis was used to test the sample differences. NS = not significant, *P<0.05, **P<0.01. Note: One replicate was used for 0.75% CHAPS sample group due to technical issues.


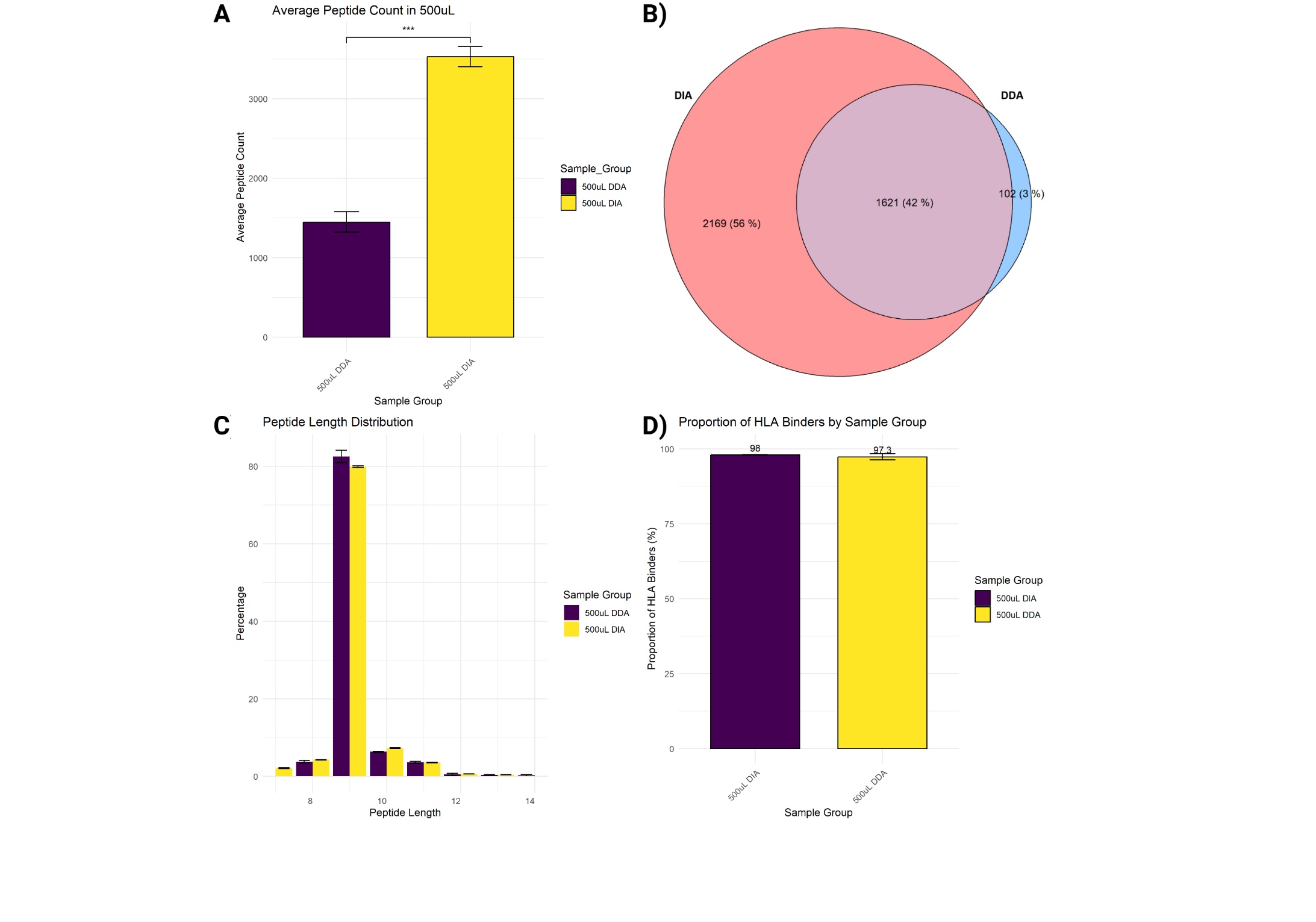


**Supplementary Figure 3. Peptidome comparison of DIA and DDA on 500 µL plasma.** **A)** vDIA outperforms DDA in peptide identification. **B)** DIA captures most of the peptides identified in DDA., in addition to significantly more peptides. **C)** Length distribution of identified peptides in each group follows the typical HLA-I peptide length distribution. **D)** NetMHCpan4.1 binding analysis. Data were acquired using 3 replicates and plotted as Mean ± SEM. Unpaired t-test was conducted to test for statistical differences in peptide count. ***P <0.001


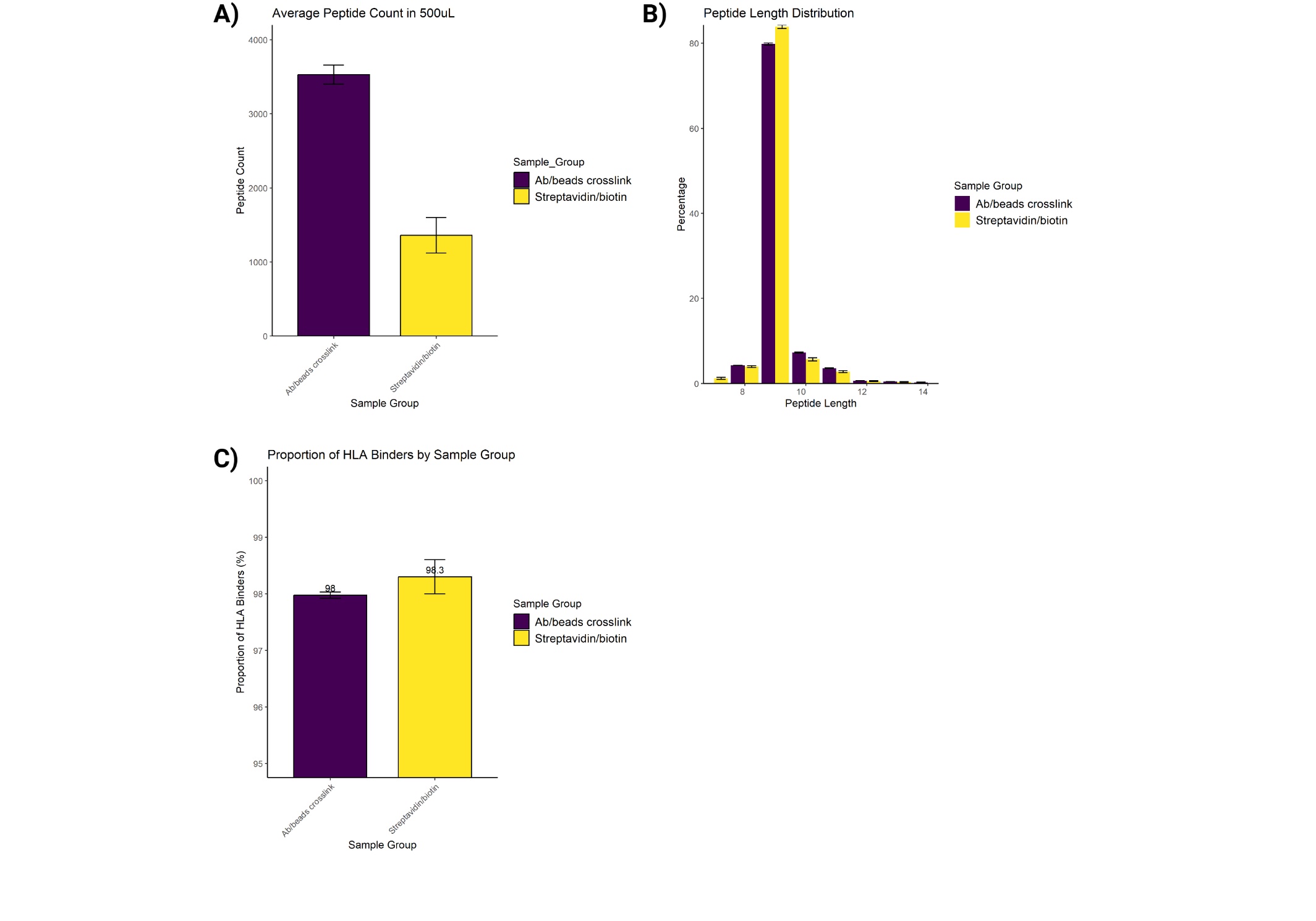


**Supplementary Figure 4. Immunopeptidome comparison of SAPrIm 2.0 and streptavidin-biotin system on 500 µL plasma.** **A)** Crosslinking antibody/beads system employed in SAPrIm 2.0 outperforms streptavidin-biotin system. **B)** Peptide Length Distribution of identified peptides. **C)** NetMHCpan4.1 binding analysis. Unpaired t-test statistical analysis was performed to test for statistical significance. ** P<0.01.


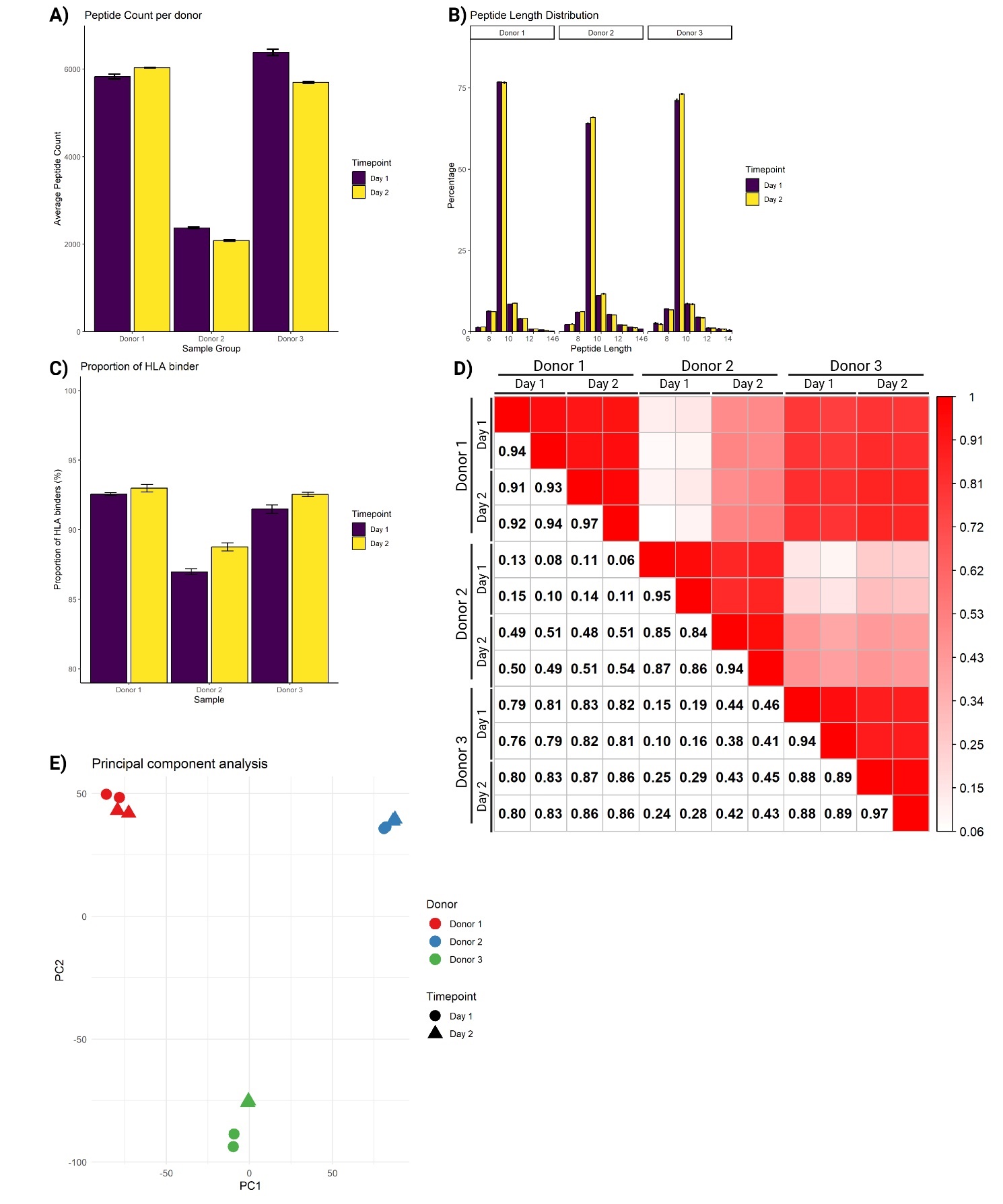


**Supplementary Figure 5. Inter-assay evaluation of SAPrIm 2.0 on 500 µL plasma. A)** Peptide identification, **B)** Peptide length distribution, and **C**) Proportion of HLA binder by NetMHCpan-4.1 remains stable across two separate experimental runs. **D)** Pearson’s correlation analysis on peptide intensity shows high correlation between samples sourced from the same donor across 2 different timepoints, but modest correlation between samples sourced from different donors. **E)** Principal component analysis shows tight clustering of immunopeptidome sourced from the same donors, and highlights distinct separation of immunopeptidome from different donors.
